# The Cause of Diabetes

**Published:** 1900-07-14

**Authors:** 


### THE CAUSE OF DIABETES.

Diabetes mellitus may arise from several causes. It has been shown by experiments that it can be produced by lesions of the fourth ventricle and of many parts of the nervous system; by removal of the pancreas, and by the administration of certain drugs. Again, by investigations made in the post-mortem-room, it is found that it is in some cases associated with, and perhaps caused by, changes in the nervous system, pancreatic lesions, and changes in the circulatory system. But it is to be admitted that in a very large number of cases neither the history of the case nor the conditions discovered after death give any clue to the origin of the disease. Dr. R. T. Williamson,[1] in discussing the cause of diabetes, urges several facts pointing to the probability of the whole thing starting from some toxic substance being introduced into the system from the intestinal canal. First he points to the rapidity with which in some cases, especially in young adults or children, the disease runs its course, the patients having been in good health up to the attack, and the onset of the symptoms having been very sudden. Then the tendency which the disease sometimes shows to intermit, and may be to disappear entirely for a time, and the influence which an intercurrent disease, such as phthisis, or of febrile attacks, exercises in holding the symptoms in check have to be remembered. Finally, he says that during the last ten years several papers have been published recording the occurrence of diabetes in both man and wife, and although such cases are comparatively rare, they seem to have occurred in a proportion which is certainly greater than can be accounted for by mere coincidence, all of which favours the view that in these cases both man and wife, having been exposed to the same influence, have suffered from the same effect. Coming to experimental evidence, it is well known that a number of drugs will so disturb sugar metabolism as to produce temporary diabetes, so that it is quite in accordance with the facts of the case to suggest the absorption of a toxin as a cause of the disease. The observation, then, that diabetic urine when administered to dogs in large quantity by the mouth, or subcutaneously, produces in them glycosuria is a matter of great interest, especially in view of the fact that non-diabetic urine does not produce this effect, for it seems to show that a diabetic is excreting, and therefore presumably contains a poisonous substance of some kind. Then to show that this poison comes from the intestinal canal there is the observation made by Töpfer that in animals the subcutaneous injection of a dialysate of fæces from a diabetic subject produced glycosuria, and that the injection of a quantity of the contents of the small intestine of a diabetic patient under the skin of a rabbit did the same, while if the intestinal contents of a healthy man were employed no such result followed. Recently, again, Töpfer has shown that glycosuria can be produced in dogs by injecting into the small intestine, or even into the stomach, a portion of the intestinal contents taken from a diabetic patient, while control experiments from non-diabetic cases proved negative. The results of all these experiments is to suggest very strongly that many cases of diabetes may be caused by the absorption from the intestines of some toxic product the result of the action of micro-organisms on their contents.

[1] Practitioner, July.

